# Supplementary material for: Thermoelectric properties of topological chains coupled to a quantum dot
Source: Sci Rep. 2023 Jan 27;13:1508. doi: 10.1038/s41598-023-28491-6 (PMC9883525; doi:10.1038/s41598-023-28491-6)
Supplement: Supplementary file 1 — Supplementary Information. [file 41598_2023_28491_MOESM1_ESM.pdf]

Thermoelectric properties of topological chains coupled to a quantum dot - Supplementary Material

A. C. P. Lima[1], R. C. Bento Ribeiro[1], J. H. Correa[2], Fernanda Deus[3], M. S. Figueira[4*], and Mucio A. Continentino[1]
[1] *Centro Brasileiro de Pesquisas Físicas, Rua Dr. Xavier Sigaud, 150, Urca 22290-180, Rio de Janeiro, RJ, Brazil*
[2] *Universidad Tecnológica Del Perú, Nathalio Sanchez, 125, Lima, Perú*
[3] *Rio de Janeiro State University, Rodovia Presidente Dutra km 298, 27537-000, Resende, RJ, Brazil and*
[4*] *Instituto de Física, Universidade Federal Fluminense, Av. Litorânea s/N, CEP: 24210-340, Niterói, RJ, Brasil*

Topological one-dimensional superconductors can sustain in their extremities zero energy modes that are protected by different kinds of symmetries. The observation of these excitations in the form of Majorana fermions is one of the most intensive quests in condensed matter physics. In this work we are interested in another class of one dimensional topological systems, namely topological insulators. These also present symmetry protected end modes with robust properties and do not require the low temperatures necessary for topological superconductivity. We consider a device in the form of a single electron transistor coupled to the simplest kind of topological insulators, namely chains of atoms with hybridized *sp* orbitals. We study the thermoelectric properties of the device in the trivial, non-trivial topological phases and at the quantum topological transition of the chains. We show that the electrical conductance and the Wiedemann-Franz ratio of the device at the topological transition have universal values at very low temperatures. The conductance and thermopower of the device with diatomic *sp*-chains, at their topological transition, give direct evidence of fractional charges in the system. The former has an anomalous low temperature behavior, attaining a universal value that is a consequence of the double degeneracy of the system due to the presence of zero energy modes. On the other hand, the system can be tuned to exhibit high values of the thermoelectric figure of merit and the power factor at high temperatures.

I. SSH AND SP-CHAIN MODELS

The Hamiltonian describing the monoatomic sp-chain is given by [1, 2],

$$\begin{aligned}\mathcal{H}_{sp} &= \epsilon_s^0 \sum_j c_j^\dagger c_j + \epsilon_p^0 \sum_j p_j^\dagger p_j - \sum_j t_s (c_j^\dagger c_{j+1} + c_{j+1}^\dagger c_j) \\ &+ \sum_j t_p (p_j^\dagger p_{j+1} + p_{j+1}^\dagger p_j) + V \sum_j (c_j^\dagger p_{j+1} - c_{j+1}^\dagger p_j) \\ &- V^* \sum_j (p_j^\dagger c_{j+1} - p_{j+1}^\dagger c_j) \end{aligned} \quad (1)$$

where $\epsilon_{s,p}^0$ are the centers of the s and p bands, respectively. The $t_{s,p}$ represent the hopping of *spinless* electrons to neighboring sites in the same orbital and V the antisymmetric hybridization between s and p states in neighboring sites. Due to the different parities of the orbital states, this hybridization is odd-parity, such that, in momentum space $V(-k) = -V(k)$. Then the mixing term breaks the parity symmetry of the system in spite that the chain is centro-symmetric. Notice that the spin-orbit coupling (SOC) also breaks parity symmetry [3], but differently from hybridization that mixes quasi-particles with the same spin, SOC mixes quasi-particles with opposite spins [3]. For simplicity we consider here the case of spinless fermions and take the chemical potential $\mu = 0$. We considerer symmetric bands, such that, $\epsilon_s^0 = -\epsilon_p^0 = \epsilon$ and assume $t_s = t_p = V = t$.

The frequency dependent Green's function G_{00} at the edge of the semi-infinite chain can be obtained as [4],

$$G_{00}(\omega) = \frac{1}{\omega}\left[\tilde{\omega}^2 - \tilde{\epsilon}^2 + 1 \pm \sqrt{(\tilde{\omega}^2 - \tilde{\epsilon}^2 + 1)^2 - 4\tilde{\omega}^2}\right] \quad (2)$$

where $\tilde{\omega} = \omega/2t$ and $\tilde{\epsilon} = \epsilon/2t$. Comparing this equation with Eq. 9 of the main text, we obtain the formal relation between the SSH and *sp*-chain models. Notice the factor (1/2) difference that appears in Eq. 9 since for the *sp*-chain the full bands correspond to two electrons per site.

II. THE *sp*-HYBRID DIATOMIC CHAIN

The Hamiltonian of the *sp*-hybrid, diatomic chain is given by [5],

$$\begin{aligned} H &= \sum_n \epsilon(n)(\alpha_n^+ \alpha_n + \beta_n^+ \beta_n) + \sum_n V_1(n)(\alpha_n^+ \beta_n + \beta_n^+ \alpha_n) \\ &+ V_2 \sum_n (\alpha_n^+ \beta_{n+1} + \beta_{n+1}^+ \alpha_n) \end{aligned} \quad (3)$$

where α_n^+ creates an electron in orbital a for n even and in orbital b for n odd. The operator β_n^+ creates an electron in orbital $\underline{a}$ or $\underline{b}$ for n even or odd respectively. $\epsilon(n)$ takes values $+\epsilon$ and $-\epsilon$ for even (a) and odd (b) sites, respectively, as shown in Fig. 1. Here we take $V_1(n) = V_1$ independent of the site to obtain a Hamiltonian similar to the Rice-Mele model. The constant terms V_2 connect orbitals $\underline{a} - b$ and $\underline{b} - a$ in different sites.

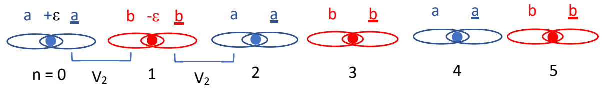

FIG. 1. (Color online) Diatomic sp-chain with similar topological properties of the RM model,

III. DATA AVAILABILITY

The data that support the findings of this study are available from the corresponding author upon reasonable request.

IV. REFERENCES

[1] E.-N. Foo and H.-S. Wong, Phys. Rev. B **9**, 1857 (1974).
[2] M. A. Continentino, H. Caldas, D. Nozadze, and N. Trivedi, Phys. Lett. Sect. A Gen. At. Solid State Phys. **378**, 3340 (2014), arXiv:1405.4183.
[3] H. Caldas, R. L. S. Farias, and M. Continentino, Phys. Rev. A **88**, 023615 (2013).
[4] E. N. Foo and L. G. Johnson, Surf. Sci. **55**, 189 (1976).
[5] E.-N. Foo and D. Giangiulio, Physica B+C **84**, 167 (1976).
